# Translating group programmes into online formats: establishing the acceptability of a parents’ sex and relationships communication serious game

**DOI:** 10.1186/s12889-015-2545-0

**Published:** 2015-12-09

**Authors:** Julie E. Bayley, Katherine E. Brown

**Affiliations:** Faculty of Health and Life Sciences, Coventry University, Coventry, UK; Centre for Technology Enabled Health Research, Coventry University, Coventry, UK

**Keywords:** Translation, Impact, Implementation, Parents, Sex and relationships, Communication, Teenage pregnancy

## Abstract

**Background:**

With ongoing concerns about the sexual health and wellbeing of young people, there is increasing need to innovate intervention approaches. Engaging parents as agents to support their children, alongside capitalising on increasingly sophisticated technological options could jointly enhance support. Converting existing programmes into interactive game based options has the potential to broaden learning access whilst preserving behaviour change technique fidelity. However the acceptability of this approach and viability of adapting resources in this way is yet to be established. This paper reports on the process of converting an existing group programme (“What Should We Tell the Children?”) and tests the acceptability within a community setting.

**Methods:**

Translation of the original programme included selecting exercises and gathering user feedback on character and message framing preferences. For acceptability testing, parents were randomised to either the game (*n* = 106) or a control (non-interactive webpage) condition (*n* = 76). At time 1 all participants completed a survey on demographics, computer literacy and Theory of Planned Behaviour (TPB) items. Post intervention (time 2) users repeated the TPB questions in addition to acceptability items. Interviews (*n* = 17) were conducted 3 months post intervention to gather qualitative feedback on transfer of learning into real life.

**Results:**

The process of conversion identified clear preferences for first person role play, home setting and realistic characters alongside positively phrased feedback. Evaluation results show that the game was acceptable to parents on cognitive and emotional dimensions, particularly for parents of younger children. Acceptability was not influenced by baseline demographics, computer skills or baseline TPB variables. MANOVA analysis and qualitative feedback suggest potential for effective translation of learning into real life. However attrition was more likely in the game condition, potentially due to feedback text volume.

**Conclusions:**

A manualised group programme can be viably converted into a serious game format which is both cognitively and emotionally acceptable. The intervention may be more effectively targeted at parents with younger children, and further game developments must particularly address information dosing. Establishing the viability of digitally converting a group programme is a significant step forward for implementation focused research.

## Background

Despite slow progress in reducing under 18 conceptions in the UK in the early 21^st^ century [1, 2] 54 % fewer under 18 s now conceive each year in England for example, compared with 18 years ago [1, 3]. Associated negative health and social consequences of teenage pregnancy [4, 5] continue to present a public health concern however; this data is included as one of three Government indicators of population sexual health [6] and UK rates of teenage pregnancy remain the highest in Western Europe [3]. In parallel, 15–24 year olds continue to have the highest rates of Sexually Transmitted Infections (STIs), forming the majority of chlamydia, gonorrhoea and genital warts cases diagnosed at UK Genito-Urinary Medicine (GUM) clinics [7]. Effort must therefore be maintained and interventions extended to support a stronger downward trend.

Tackling teenage sexual health is complicated by the breadth of factors which inhibit safe sex practices. Research evidence highlights the significant effects of: (i) environmental and familial influences [8], ii) interactional and situational variables [9, 10] and (iii) adolescent-specific cognitive processes [11, 12], the coalescence of which may increase the likelihood of risky behaviour. Increasingly educators are also seeking to inculcate skills to resist sexual pressure and engender positive relationships [13]. The success of safer sex interventions therefore is tempered by their ability to effect change in this complex ecological system [14].

Increasingly best practice in public health is underpinned by drives to embed behaviour change theory into programmes [15]. However, brief interventions struggle to counteract the combined and deep-rooted influences on safer sex behaviour [16, 17]. Outcomes tend to be more positive where interventions are more intensive and theory driven [18, 19], but the resource demands of facilitated approaches [20] may prohibit such approaches. Thus there is need to innovative methods for providing theory-rich, individualised support.

Despite international evidence of the positive impact of school based sex education, provision remains patchy within the UK [21]. Accordingly the value of engaging parents as educators – in addition to targeting young people directly - is crucial and has been strategically recognised [22]. Good parent–child dialogue on sex and relationships (SR) is associated with reduced likelihood of unsafe sex [23, 24]. Systematic review evidence suggests that parent communication interventions can yield improvements in frequency, quality and comfort of parent–child SR communication [25]. Endpoints of research in this area tend to concur on the need to further innovate parental communication programmes and more broadly disseminate best practice to extend their reach [25, 26].

“What Should We Tell the Children?” (WSWTTC [27]) - a group based parents’ SR communication programme co-devised by the lead author – was developed to enhance the availability of theory and evidence based training. Using an Intervention Mapping approach [28], WSWTTC was created through an iterative process based fundamentally on users’ (parents’) needs and incorporating published evidence, theory and stakeholder expertise. The programme was (and continues to be) delivered as a facilitated multiple session group course. However, despite pilot testing showing benefits to attitude [29], WSWTTC has faced both practical difficulties (e.g. venue costs) and reluctance to engage from certain sub-sections of the population (e.g. fathers [30]). Thus, the programme had restricted reach and needed innovating to overcome barriers to engagement.

Online approaches offered the potential to remove practical barriers and offered a less daunting mode of learning for those less willing to attend group sessions. More specifically serious games offer learning in a more entertaining format whilst preserving interactivity and content fidelity. SGs create more lifelike examples and help transpose learning more readily into real-world experience via exploratory and situative learning [31]. Evidence suggests that SGs are an effective and efficient means of delivering targeted behavioural outcomes with longer lasting effects [32]. They also reflect the broader appeal of electronic gaming to the general public [33] and the growing body of technology-savvy ‘digital natives’ [34].

Medical Research Council guidance [35] on the development of complex behavioural interventions advocates that programmes are built “*using a carefully phased approach, starting with a series of pilot studies targeted at each of the key uncertainties in the design”* (pg.8). Ahead therefore of a more sizeable research programme, it is essential to establish whether an online SR communication game would be acceptable to the target audience. Additionally the viability of converting existing programmes must be established, as despite the potential public health benefits such innovations may proffer little is known about the conversion process. This study explores the feasibility of translating a traditional group programme into a game format and examines the acceptability of the game itself. The overall aim was to establish whether a manualised group programme can be viably converted into an acceptable game format to improve parents’ SR communication. Specifically, the paper aimed to assess whether:

i. a game format is acceptable to parents
ii. game acceptability is influenced by underlying demographic variables, computer literacy levels or psycho-social variables
iii. the game demonstrates the potential to effectively change attitudes, intentions and behaviour relating to parental SR communication

## Methods

### Original intervention

The WSWTTC group programme [27] consisted of six facilitated sessions including multiple exercises on initiating conversation, capitalising on opportunities for discussion and responding effectively to children’s questions. With the needs assessment showing attitudes, self efficacy and knowledge to be determinants of communication, content and exercises were devised via Intervention Mapping [28] to target and improve these.

### Translation into gaming format

Components from the WSWTTC group programme were reviewed for potential conversion based on parent acceptability, usefulness, viability of conversion into a gaming format and adherence to the original intervention map. Exercises chosen for conversion were those which satisfied the following conditions:

- Original exercise was well received and valuable in group based programme (*facilitator judgement*)
- Convertible into first-person role play (*developer judgement*)
- Mapped against key elements of the original intervention map (*researcher judgement*)

A total of five scenarios plus a quiz were identified for conversion covering the skills of responding effectively to child queries, initiating difficult conversations and building openness in communication for future discussions. A comparison of the original programme structure and selected conversion into the game is given in Table 1.

**Table 1:** Original WSWTTC content and conversion details

Group Session	Exercise title	Summary	Conversion into game element
1: Is there more to it than the birds and the bees?	Birds and the bees and much more	Group discussion to identify topics within RS to increase parents’ understanding of breadth of SR	Not converted
	Tree	Graphic representation (tree) to enable parents to visualise and track progress on the course	
	My job description	Small group discussion/individual work to develop a ‘job description’ for their unique role in SR communication.	
	Vocabulary	Group discussion of SR vocabulary to improve parents understanding, comfort and confidence in using appropriate terminology	
2: Is there a right time to talk about it?	My plan	Small group discussion and individual planning of age appropriate communication with children.	
	Identifying opportunities	Group discussion to identify opportunities for/increase confidence in initiating SR communication in everyday life.	
3: What do I say when I’m put on the spot?	Story	Story about children’s reaction to poor school sex education and increase parents’ understanding that ineffective communication can lead to children seeking out answers from less reliable sources.	Scenario 1: Child asks parent to explain a documentary in which lions are mating *(Story replaced with TV programme to make visual and home-based).*
	Basket of items	Group activity: parents pick an item from a box (e.g., condoms, bullying message on social media, adult magazine) and give their reaction as if they found this in their child’s room. Objectives include developing parents’ skills and confidence in responding calmly and effectively	Scenario 2: Parent finds variety of items (e.g., sexualised magazine, social media messages) in child’s room (*Box changed to virtual bedroom*)
4: What do I say and will they take any notice?	Considering my message	Individual and group activity to help parents develop clear values/messages regarding SR	Scenario 3: Child asks parents about same sex relationships
	Improving your communication style	Role play: parents (acting as child) ask questions to the facilitator (acting as parent) to consider the effect of both bad and good communication.	Scenario 5: Child discusses emerging feelings for someone at school (*Question is asked by child rather than to facilitator*)
5: Can I do this and still protect their innocence?	Risk and protection quiz	Multiple choice quiz to provide accurate information on (e.g.) adolescent sexual activity and children’s preference for parental communication.	Quiz: Quiz show rounds between scenes (*Change from paper based quiz to game show format*)
	Advice column	Group discussion using real ‘agony aunt’ questions, the group discusses the answers given and how their responses would differ.	Scenario 4: Child asks parents why they argue. (*Change from agony aunt questions to direct questions from children*)
6: Can I do this without it being embarrassing?	Role play	Consolidate and practice knowledge/skills developed	Not converted
	Action plan	Plan long term implementation of learning into the home	

### Characters and images

A range of character options and backgrounds were designed by the technical team, including both realistic and cartoon-style images. These were reviewed by 17 parents within two parenting groups, and a clear preference emerged for first person role play (*n* = 16), a home setting (*n* = 14) with realistic characters (*n* = 16).

### Game script

Response options were based on a thematic analysis during the needs assessment [27] through which parents were found to react to children’s questions in one of three main ways: (i)*functional* (parent opens up conversation and the child optimises understanding), (ii)*avoidant* (parent refuses to answer or changes the subject), or (iii)*overreaction* (parent jumps to conclusions or reacts overly strongly, leading to the child becoming angry or disengaged). A branched dialogue script was developed, wherein the child responses depend on the parents’ reactions.

### Message framing of feedback text

To mirror group discussions in the original programme, feedback text was constructed to offer parents insight into the effect of their communication choices. An online survey with 62 parents explored preferences for the framing of these messages in terms of positive/negative reinforcement, numeric feedback style and extent of evaluation statements. Results (see Table 2 for feedback options) showed an overwhelming preference for positively framed questions (100 %), percentages (45.9 %) and evaluation statements with targeted questions (35.2 %). Game feedback text was then constructed accordingly.

**Table 2:** Parents’ message framing preferences

Framing component	Option types	Example	% pref
Positive vs. negative messages	Positive	If you talk with your children about sex and relationships, they will be better able to deal with difficulties	100 %
	Negative	if you don’t talk with your children about sex and relationships, they will be less able to deal with difficulties	0 %
Numeric feedback (summary at end of game)	Percentage	You respond positively to your child 80 % of the time.	45.9 %
	Fraction	You respond positively to your child four-fifths of the time	0 %
	Ratio	You respond positively to your child four out of five times.	16.4 %
	Proportion	You respond positively to your child more than three quarters of the time	3.3 %
	General	You respond positively to your child most of the time	34.4 %
Evaluation statements	Statement only	You generally react negatively to difficult or embarrassing situations	11.1 %
	Statement + interpretation	You generally react negatively to difficult or embarrassing situations, probably because you don’t feel able to deal with them	3.7 %
	Statement + suggested changes	You generally react negatively to difficult or embarrassing situations, and you need to think about how to understand things from your child’s perspective	24.1 %
	Statement + targeted questions	You generally react negatively to difficult or embarrassing situations. Is this because you don’t know what to say? You don’t have the confidence? Or is it something else. Think about what is making you react so strongly at times	35.2 %
	Statement + reflection from child’s perspective	You generally react negatively to difficult or embarrassing situations because you find it difficult to know how to respond. As a result you respond quickly, sometimes without finding out more information from your child first. You should think about how this impacts on your child	25.9 %

A summary of the final game is given in Table 3.

**Table 3:** “What Should We Tell the Children?” game summary


The game provides parents with realistic scenarios of sex and relationships communication with their children, all based around a virtual house. Once registered, players are then able to select from two versions of the game – one for parents of younger children (aged 5–9) and one for older (aged 10–14). Both versions have similar content and the same gameplay, with slight dialogue differences to reflect the nature of conversations at different ages. The game is a first-person role play, with players proceeding through rooms of a virtual house and talking to ‘their children’. In each room they are faced with a different situation such as children asking awkward questions or finding objects of concern in their room (e.g., messages on social networking sites). Scenarios include:
1. Child asks parent to explain a documentary in which lions are mating.
2. Parent finds variety of items (e.g., sexualised magazine, social media messages) in child’s bedroom
3. Child asks parents about same sex relationships
4. Child asks about parents arguing
5. Child discusses emerging feelings for someone at school
In each situation, the player must choose how to respond, and the scenario evolves accordingly with the child reacting to the parents’ choices. Scenes are interspersed with short quizzes to increase knowledge and raise awareness of key issues. Players receive feedback on their choices at the end of each scene and full feedback at the end of the game with tailored advice on how to improve their skills. Voice-overs for the child characters and atmospheric music were added to make the scenarios more engaging. All spoken text was displayed on screen so the game can be played with or without sound. The game takes approximately 1 h to complete, but can be played at the parent’s chosen pace.
The game can be viewed at https://healthinterventions.coventry.ac.uk/sash/-projects-parents-game.aspx

### Control condition

A non-interactive webpage version was produced as an active control, comprising the messages framed in the same way, but without interactive gameplay or tailoring of feedback.

### Participants and procedure

The study was conducted in Coventry and Warwickshire (Midlands, UK) and ethics approval was given by Coventry University Ethics Committee. The game was marketed widely across the region, using established channels of public health marketing (print media, radio, posters, existing parenting groups and via major employers). Eligibility was restricted to over 18 year olds, parental responsibility for at least one child under 16 (no minimum age set to allow for those seeking to prepare for later conversations), access to the internet and the ability to read and understand English. All aspects of the study - including Participant Information and Consent processes - occurred online, accessible from any computer with internet access. Self-selecting parents visited the website, registered to participate and were then automatically randomised to the experimental or control condition. Participants completed baseline (T1) and follow up measures (T2) in a single sitting, with a subset also providing interview data at three months post intervention.

### Measures

The needs assessment in the original programme [27] determined that the Theory of Planned Behaviour [36] most clearly matched the identified psychological determinants of sex and relationships (SR) communication. This socio-cognitive model posits behaviour as a direct function of intention, which itself is derived from Attitude (ATT; belief in the value of an action), Subjective Norms (SN; beliefs about how others think they should behave) and Perceived Behavioural Control (PBC; confidence in ability to perform the activity). These factors were therefore targeted in the original programme and thus the game. TPB survey items were devised according to recommended practice [37, 38]. Each TPB construct was measured through a series of 7 point Likert scales (1 = strongly disagree to 7 = strongly agree). Intention to talk with children about SR was computed from two items *(“I intend to talk with my children”* and *“I want to talk with my children”*). PBC was derived from two items *(“if I wanted to I could talk with my children”* and *“It is mostly up to me whether or not I talk with my children”*). SN was computed through two standard normative items *(“People who are important to me think I should*” and *“People who are important to me talk to their children”*) plus one moral norm item (*“I should talk with my children”*). ATT was calculated from four semantic differential items (*“Talking with my children about SR is” (i) Harmful/helpful, (ii) Bad/good, (iii) Not important/important, (iv) Embarrassing/not embarrassing*) in which scores reflect the extent of conceptual agreement with the term. In all cases higher scores indicated more agreement / positive levels of the construct. Mean scores were calculated for each scale and TPB scales showed good internal reliability. Cronbach’s alpha levels were as follows: Intention (2 items, α = .885), Attitude (4 items, α = .879), Subjective Norm (3 items, α = .767), PBC (2 items, α = .708). Analysis showed that removing the moral norm item marginally improved SN scale reliability (to .780), but given the small difference and contextual relevance of this item it was retained. A measure of behavioural frequency was not included because (i) piloting in the original programme identified difficulty developing a meaningful measure of episodic, context-based dyadic behaviour, and (ii) as the intervention and pre-post measures were conducted in a single sitting it was not possible to capture actual behaviour change in that time. A single sitting design was chosen to maximise likelihood of engagement and survey completion. Acceptability of both the game and control version were measured by a series of 7 point Likert scale items (strongly disagree to strongly agree) covering ease of use, usefulness, willingness to recommend the resource to others and discomfort/anxiety using the resource. These items were factor analysed to determine the underlying elements of acceptability. Real-life similarity of characters, setting, and dialogue were measured on 5 point scales (*completely different* to *very similar*). Computer literacy was calculated as a product of self reported computer literacy (*not at all good* to *very competent*) and length of time using a computer (*less than 6 months* to *more than 5 years*).

At baseline (Time 1, immediately before intervention) all participants completed measures on demographics (gender, ethnicity, age, number of children, child’s ages), computer literacy (length of computer use, self rating of literacy), frequency of parent website access and direct TPB measures (ATT, SN, PBC, INT). At Time 2 (immediately post intervention), all participants repeated the TPB measures plus acceptability items for the relevant condition.

Telephone interviews were conducted three months post intervention to gather data on the translation of learning into real life settings, longer term impact and overall acceptability. All game participants were asked to consent at T1 to being contacted for a follow up phone interview. Those who consented were sent an email invite, followed by one repeat request to non responders, and no further contact was made for those not responding after this second invitation.

### Data analysis

Chi square tests were used to assess relationship between attrition and condition (game vs. static control), demographics and computer literacy. Factor analysis was run to determine underlying components within measures of acceptability. The influence of underlying demographic, computer literacy or TPB variables on acceptability was assessed with linear regressions. MANOVA analysis was undertaken to assess whether game acceptability differed by gender, ethnicity or child’s age and to assess the effectiveness of the game on TPB variables.

All analysis was run on the dataset with missing T2 data and again replacing missing data with T1 scores (Intention to treat analysis) but no differences were found. All results presented here are without replaced data.

## Results

A total of 180 registered for the study and completed a time 1 survey. Ages ranged from ‘under 20’ (10 %) to ‘61 and over’ (1.1 %) with a normal distribution (mode = 31–35 years old). The majority (83.3 %) were female, and most were White British (88.3 %). Sample characteristics are presented in Table 4.

**Table 4:** Participant demographics

Total	T1		T2	
Demographics	*N*	%	*N*	%
Age				
Under 20	18	10.0	7	7.5
21–25	15	8.3	5	5.4
26–30	15	8.3	10	10.8
31–35	37	20.6	21	22.6
36–40	34	18.9	15	16.1
41–45	29	16.1	15	16.1
46–50	15	8.3	9	9.7
51–55	12	6.7	7	7.5
61+	2	1.1	2	2.2
(Missing)	3	1.7	2	2.2
Ethnicity				
White British	159	88.3	80	86.0
White other	6	3.3	5	5.4
Indian (Asian/British Asian)	8	4.4	5	5.4
Bangladeshi (Asian/British Asian)	3	1.7	1	1.1
Pakistani (Asian/British Asian)	2	1.1	2	2.2
Asian other/Asian mixed	1	0.6	0	0
Mixed Heritage	1	0.6	0	0
Number of children				
1	71	39.4	40	43.0
2	77	42.8	42	45.2
3 or more	32	17.8	11	11.8
Child’s age				
Pre-School/Primary	107	59.4	58	62.4
Secondary/over	43	23.9	24	25.8
Both	19	10.6	8	8.6
(Missing)	169	93.9	3	3.2
Length of time using computer				
Less than 6 months	6	3.3	3	3.2
Between 6 months and 1 year	6	3.3	3	3.2
1–5 years	11	6.1	4	4.3
More than 5 years	157	87.2	83	89.2
Frequency accessing online resources				
At least once per day	18	10.0	8	8.6
At least once per week	21	11.7	11	11.8
At least once per month	32	17.8	15	16.1
Less than once per month	65	36.1	33	35.5
Never	44	24.4	26	28.0
Computer literacy				
Not at all good	9	5.0	6	6.5
Below average	1	0.6	1	1.1
Average	27	15.0	11	11.8
Competent	55	30.6	28	30.1
Very competent	88	48.9	47	50.5
Gender				
Female	150	83.3	80	86.0
Male	30	16.7	13	14.0

Table 5 shows the mean scores and standard deviations for TPB constructs at baseline and at time 2.

**Table 5:** Mean and standard deviation scores for TPB constructs at T1 and T2

Construct	GAME		CONTROL	
	T1	T2	T1	T2
INT	6.25 (1.14)	6.33 (1.25)	5.86 (1.38)	5.81 (1.52)
ATT	6.01 (1.22)	6.16 (0.99)	5.71 (1.57)	6.14 (1.10)
SN	5.64 (1.08)	5.70 (1.35)	5.41 (1.33)	5.54 (1.46)
PBC	5.92 (1.21)	6.22 (1.27)	5.57 (1.48)	5.63 (1.58)

### Retention and attrition

**Fig. 1:**
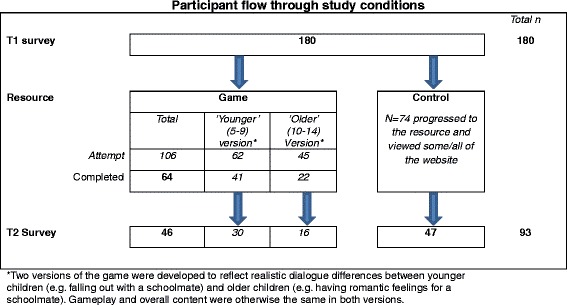
Participant flow through study conditions. *Two versions of the game were developed to reflect realistic dialogue differences between younger children (e.g., falling out with a schoolmate) and older children (e.g., having romantic feelings for a schoolmate). Gameplay and overall content were otherwise the same in both versions

Figure 1 summarises participant flow. In the game condition, of 106 participants at T1, 64 completed the game and 46 completed the T2 survey (overall attrition rate of 56.6 %). In the control condition, of 74 participants at T1, 47 viewed the resource and completed the T2 survey (attrition rate of 36.5 %).

Chi square tests were performed to identify if attrition was related to condition, demographics (gender, age, ethnicity, child’s age) or computer literacy. Results showed a significant association between condition and attrition only (*χ2* (1, *N* = 180) = 7.06, *p* = .006), showing those in the game condition were more likely to dropout before completing T2. Further chi squares showed no significant relationship between attrition and any other variable (all *p*s > .05). Participant dropout was therefore unrelated to participant characteristics or computer literacy.

### Research question 1: Is a game format acceptable to parents?

**Fig. 2:**
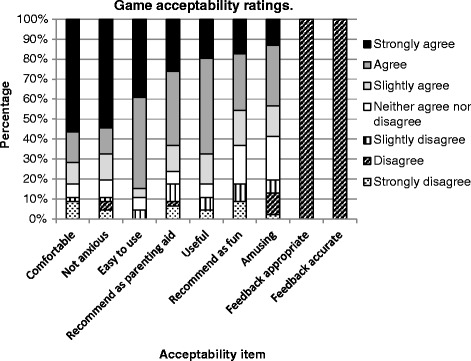
Game acceptability ratings

Descriptive data shows the game was acceptable to participants (See Fig. 2).

However, accuracy and appropriateness of feedback text was uniformly disliked. These items were excluded from subsequent factor analysis due to the lack of variance. The majority of scores also showed good similar/very similar ratings for environment (63 %), character (65.2 %), dialogue (69.6 %) and situations (63 %).

### Research question 2: Is game acceptability influenced by underlying demographic variables, computer literacy levels or psycho-social variables?

Factor analysis of the acceptability items identified underlying elements of acceptability. Kaiser-Meyer-Olkin (KMO) coefficient scores (0.773) and Bartlett test of sphericity (0.00) indicated satisfactory confidence for the execution of factor analysis. Principal components analysis with varimax rotation was applied to the data. The number of retained factors was defined based on components with eigenvalues higher than 1 and scree plot visualisation. Two factors with eigenvalues ≥1.0 were retained and the inflexion of the scree plot confirmed two factors. Analysis of the conceptual meaning of these factors was independently assessed by each author with clear agreement on the concepts. Two clear factors (acceptability types) emerged (See Table 6):

**Table 6:** Game factor analysis loadings

Game acceptability	Mean	SD	Factor 1	Factor 2
I found the game useful	5.50	1.44	.895	-
I found the game amusing	4.80	1.63	.805	-
I would recommend the game to others as a parenting aid	5.33	1.74	.887	-
I would recommend the game to others as a fun experience	4.91	1.72	.884	-
I was anxious/worried playing the game (*reverse scored*)	5.78	1.74	-	.826
I was uncomfortable playing the game (*reverse scored*)	5.83	1.83	-	.848

- Factor 1: *Cognitive acceptability* (items loading included; useful, amusing, recommend as parenting aid, recommend as fun)
- Factor 2: *Emotional acceptability* (items loading included; anxious/worried, uncomfortable – *NB Items reverse scored, higher scores showing less anxiety / discomfort*)

Linear regression analysis was conducted to determine whether game acceptability was predicted by underlying demographic, computer literacy or TPB variables. Gender, ethnicity, age, child’s age, computer use and TPB variables were regressed onto Cognitive (Factor 1) and Emotional acceptability (Factor 2). All results were non-significant (*p* > .05) with no variables predicting scores on either factor. Only ‘frequency of accessing parenting materials’ approached significance for Factor 1 (Table 7) and subjective norm for Factor 2 (Table 8).

**Table 7:** Linear regression results for cognitive acceptability of game (Factor 1)

Constant	B	Standard error	β	*P*-value
TPB				
Attitude (T1)	.172	.183	.152	.352
PBC (T1)	.142	.257	.117	.586
Subjective norm (T1)	.303	.294	.208	.310
Intention (T1)	-.309	.295	-.200	.301
Demographics				
Age	-.133	.147	-.166	.370
Number of children	-.204	.224	.-.143	.368
Computer literacy/use				
Computer literacy	.305	.249	.189	.228
Frequency accessing online parenting resources	-.379	.197	-.298	.062

**Table 8:** Linear regression results for emotional acceptability of game (Factor 2)

Constant	B	Standard error	β	*P*-value
TPB				
Attitude (T1)	-.268	.208	-.219	.204
PBC (T1)	.060	.293	.206	.838
Subjective norm (T1)	.612	.335	.390	.075
Intention (T1)	-.524	.335	-.314	.126
Demographics				
Age	-.127	.167	-.146	.453
Number of children	-.018	.255	-.012	.943
Computer literacy/use				
Computer literacy	-.151	.284	-.087	.597
Frequency accessing online parenting resources	.201	.224	.147	.374

Further analysis was conducted to assess whether game acceptability differed by gender, ethnicity or the child’s age. MANOVA results showed a significant effect of child’s age on Cognitive Acceptability (Factor 1), (*F* (2, 38) = 3.764, *p* = .032; partial η^2^ = .165). Post hoc univariate analysis revealed that cognitive acceptability was significantly higher for parents of young children, (*F*(2, 43) = 3.436, *p* = .0141; partial η^2^ = .138). Results for ethnicity and gender were not significant, and no effects were found for Factor 2 (emotional acceptability).

Data suggests acceptability is not predicted by underlying TPB cognitions or computer familiarity, but that being a frequent user of existing online parenting resources may be related to cognitive acceptability of the game.

### Research question 3: Does the game demonstrate the potential to effectively change attitudes, intentions and behaviour relating to parental SR communication?

To assess the likely effectiveness of a fully powered intervention on TPB constructs, a 2x2 (Time*condition) MANOVA was conducted. Results showed a small main effect of time only (*F* (4, 88) = 2.515, *p* = .047; partial η^2^ = .103). Within subjects univariate tests showed a change in attitude by time (F(1, 91), = 6.798, *p* = .011, partial η^2^ = .07) and time*condition interaction effects approached significance for attitude (F (1,91) = 3.616, *p* = .06, partial η^2^ = .038).

Qualitative data also offers insight into the transfer of learning into real life. A total of 17 parents consented at T2 to be contacted for follow up at three months. When contacted, 13 agreed to be interviewed, 2 declined and 2 did not reply. To supplement this feedback, 4 further interviews were conducted with stakeholders who represented key gatekeepers and delivery agents. These individuals consisted of a youth worker (who works with young parents), one community worker (who delivers interventions in the local area), one Sex Education teacher (for whom the game would add to their broader engagement programme) and one public health lead (for whom the game may form part of local provision). As those working at the interface between parents/provision and commissioning, they were able to offer an aerial insight into the reception and usefulness of the course. All played the game and were interviewed 3 months post intervention. Responses (summarised in Table 9) suggest the game offers parents the means to reflect on and improve their existing communication skills. Responses indicate instances of improved skills and actual behavioural changes underpinned by changes in attitude.

**Table 9:** Summary of qualitative feedback (3 months post intervention)

1. Benefit of Serious Game Approach
• ‘Made it a bit more real than just the info you get in leaflets and books’.
• ‘I liked that fact that the situations presented were normal – watching TV, in the kitchen and so on’.
2. Changing attitudes and behaviours
• ‘It’s so easy to get yourself tied up with what you should say, what you shouldn’t say, what will other parents think and so on. This game made me think more about how I say things and I how I keep things open with my kids…It’s more important to make sure your kids can come to you and talk when they need to’.
• ‘The game I think helped most by making me realise it’s more about making sure the channels of communication are open and discussing things rather than me just deciding what they need to know and sticking with that whatever they say’
3. Increasing awareness of opportunities
• ‘It made me realise maybe I’m not even noticing opportunities to talk about sex at home. Actually there was something that day that when I got back home, if I hadn’t played the game I don’t think I’d have picked up on at all. I wouldn’t have done anything with it, it would have passed me by, a comment my daughter made. Playing the game made me think about what I could be doing more’.
4. Adjustment to own communication style:
• ‘My daughter is … starting to notice women in magazines and how glamorous/thin they are. When she was looking through a magazine I sat with her and chatted about the pictures. I wanted to shout they’re airbrushed and fake! But I knew if I did she’d stop listening right away. So I took some of the tips as my feedback suggested I tend to be a bit overkill and I took a breath and asked her what she thought, if she thought the photo might have been changed, what was beautiful about the lady, what was beautiful about other people and just chatted it through. By the end of the chat I’d made my point and I think crucially she’d felt that she’d come to that decision herself.’

## Discussion

### Summary of findings

This study primarily sought to establish the acceptability of a computer game version of a manualised group programme. Results show the game was acceptable in both cognitive and emotional terms, uninfluenced by demographics and underlying psychosocial variables. The game was most acceptable to parents of younger children, and qualitative results indicate the game has potential to support SR communication changes longer term. Overall this study suggests a manualised group programme can be viably converted into an acceptable serious game format. Establishing the viability of converting a programme in this way and the associated public acceptance is a significant step forward for implementation focused research.

### Implications for future developments

This small feasibility study identifies a range of issues which need further exploration in a larger, higher powered programme of research. Despite high acceptability of the game, attrition rates were higher in the game compared to the control condition. The length of the game, compared to the more swiftly read static version, may have led to disengagement over time and thus contributed to attrition. Data also shows that feedback text – a key difference between the intervention and control – was routinely disliked. This is likely to have been a significant contributor to dropout in the game condition. Whilst the message framing had been assessed by users, the cumulative text volume may have deterred parents from continuing. This raises questions for information ‘dosing’ and intensity in future versions with a recommendation that the overall length of the game be shortened by reducing the amount of in-game text *alongside* managing user expectations about realistic time commitment. Ultimately intervention designers must balance content volume with message necessity to produce change.

The game was more acceptable for parents of younger children, but the reasons why are unclear. A potential explanation is that - with sexual behaviour being more distal - parents of younger children feel less anxious than those of older children. Evidence suggests that procrastination can lead to long term build up of anxiety about sex and relationships communication [39]. Parents of older children may therefore feel both the pressure to communicate and the unease of doing so after previous non-communication. As such, the game may be better suited as a preparatory tool ahead of the imminent need for such conversations to build earlier family communication.

There is also a discrepancy within research question 3 between qualitative findings (which highlight the benefit of the game in changing attitudes and confidence) and quantitative results (which show no effect by condition). This is potentially a result of the process of measurement. This study was intentionally a rapid ‘one sitting’ pre-post study based on our experience of engagement with longer term programmes. However, this necessitated minimal TPB items and precluded capture of broader effects such as those emerging from the qualitative feedback. A broader, more comprehensive and longer term quantitative assessment is crucial in future research to assess the impacts beyond a reductionist theory-specific approach.

### Strengths

The study has two primary strengths. First, the conversion process and user feedback offers insight into the process of translating an existing theory based intervention into an online version. For practitioners, this demonstrates that programmes struggling to reach the desired community can be transformed transparently and preserve the underlying knowledge base. Second, the setting and methods of the study have particular ecological validity. The game was based on user needs, marketed, released and recruited using existing standard public health approaches, and used online platforms which are widely available. Besides capitalising on existing marketing options and directly contacting some of the key gatekeeping organisations to promote the work, no artificial inflation of the recruitment was attempted (for instance no incentives were offered). This allowed a more valid assessment of game uptake and highlighted needs for further rollout. The study demonstrates the potential for translating existing programmes for delivery in this format, not having to ‘start from scratch’. The mix of both qualitative and quantitative methods enabled an assessment of underlying constructs and acceptability alongside real world translation of learning and actual change in behaviour.

### Weaknesses

There are a number of study limitations, primarily resulting from the small self-selected sample and ‘one sitting’ design. Whilst the recruitment strategy simulated real world approaches, the small sample – coupled with small expected effect sizes in behavioural research [40] – have limited the ability to detect any effects. Similarly homogeneity of the sample in terms of gender, computer literacy and particularly ethnicity reduce the generalizability of the conclusions. The voluntary nature of participation also precluded assessment of what prevented people from engaging; thus we cannot determine what deterred potential users from participating. The project was designed as a prototype, but the resulting limited character options, restricted bank of dialogue choices and prescribed gameplay settings reduced the simulative benefit of SGs. Qualitative feedback implies it still had merit over static options, but the mixed ratings on similarity items suggest there is considerable opportunity to enhance the gameplay and within-game options. Additionally whilst the system was supposed to be automatically randomising, the difference in sample sizes between conditions suggests a fairly large difference in allocation. Statistical checks demonstrate no differences between groups on key variables and no other patterns were found in manual checks of the data. Thus whilst true automatic randomisation of participants is uncertain, data checks suggest no major problems with bias.

### Future research/next steps

With qualitative feedback suggesting a simulative game approach supports parents to adopt more positive communication styles, and quantitative data indicating likely influences on attitude towards communication about sex and relationships, the game has the potential to effect real change. Future iterations of the game – particularly with revisions to feedback text – could substantially enhance these emerging impacts. Development of this approach is fourfold. First, as the game is a prototype, it needs revising and extending in both content and broader gameplay experience. Whilst overall acceptability was good, the loss of participants from pre to post intervention dilutes conclusions over effectiveness. Attrition rates therefore suggest there is need to focus on gaming elements which sustain motivation to engage [41]. Mechanisms to achieve this in a non-facilitated virtual environment need further investigation. These edits and foci should strengthen the conservative positive effects suggested in this pilot study. Second, future rollouts of the programme must include a larger and more representative sample, with long term effects tested with a more powerful research design. A Cluster RCT could be particularly appropriate given the regional (cluster) nature of many public health programmes and further need to elucidate within-family dynamics [42, 43]. A study of this scale is needed to ascertain the true potential for benefits possible through this approach. Third – given the limited uptake despite extensive marketing - future rollout requires more innovative recruitment strategies. Data suggests parents with younger children are a key audience, and may benefit from targeted approaches. Finally, the process of converting manualised programmes as outlined here should be replicated across other public health interventions to improve uptake. Such approaches are crucial for improving the successful implementation of interventions beyond the academic context.

## Conclusions

A serious game translation of an existing parenting intervention is a viable and acceptable tool for public health practitioners, particularly for parents of younger children. A broader programme of research is needed to develop and test a more comprehensive game. Translating traditional formats – so crucial for improving the implementation of academic interventions – must shift beyond information provision online into engaging and technically enhanced approaches. This pilot study has demonstrated the potential for translating traditional programmes into innovative and engaging approaches and offers a procedural insight on conversion for intervention developers.

## Abbreviations

SR: Sex and relationships
TPB: Theory of planned behaviour
WSWTTC: “What Should We Tell the Children?”
